# A shared mechanism of defense against predators and parasites: chitin regulation and its implications for life-history theory

**DOI:** 10.1002/ece3.766

**Published:** 2013-11-20

**Authors:** Andrew P Beckerman, Job de Roij, Stuart R Dennis, Tom J Little

**Affiliations:** 1 Department of Animal and Plant Sciences, University of Sheffield, Western BankSheffield, S10 2TN, U.K; 2 Ashworth Laboratories, Institute of Evolutionary Biology, University of EdinburghKings Buildings, Edinburgh, EH9 3JT, U.K

**Keywords:** Chitin, disease, endocrine physiology, inducible defenses, parasites, predation, trade-offs

## Abstract

Defenses against predators and parasites offer excellent illustrations of adaptive phenotypic plasticity. Despite vast knowledge about such induced defenses, they have been studied largely in isolation, which is surprising, given that predation and parasitism are ubiquitous and act simultaneously in the wild. This raises the possibility that victims must trade-off responses to predation versus parasitism. Here, we propose that arthropod responses to predators and parasites will commonly be based on the endocrine regulation of chitin synthesis and degradation. The proposal is compelling because many inducible defenses are centered on temporal or spatial modifications of chitin-rich structures. Moreover, we show how the chitin synthesis pathway ends in a split to carapace or gut chitin, and how this form of molecular regulation can be incorporated into theory on life-history trade-offs, specifically the Y-model. Our hypothesis thus spans several biological scales to address advice from Stearns that *“Endocrine mechanisms may prove to be only the tip of an iceberg of physiological mechanisms that modulate the expression of genetic covariance”*.

## Introduction

All organisms deal with multiple forms of stress. The ability to respond to one form of stress often comes at the expense of responding effectively to another. Such trade-offs are central to understanding evolution, species coexistence, and how physiology and development structure a phenotype. The following article is focused on two important biological stressors, predation and parasitic infection, and proposes a framework for studying how defenses against these different biological enemies trade-off. We propose a mechanistic hypothesis that is centered on the synthesis and regulation of chitin, and we then intertwine this with classic life-history theory on trade-offs.

Our chitin-centered mechanistic hypothesis is made via a focus on data from arthropods, yet the importance of chitin extends beyond this group. Chitin is found in invertebrates and vertebrates, and is one of the most abundant polysaccharides in nature. In addition to being directly involved in altered molting and remodeling of body plans in arthropods, the chitin-regulatory pathway plays a role in conserved processes such as cell growth, turnover, and remodeling, which are ubiquitous in all organisms and relevant to, for example, tumor formation and growth. Chitin and chitin-like proteins are also central features of both invertebrate and vertebrate immune systems. A chitin-based hypothesis is thus specific to arthropod defenses, but also general to many other physiological trade-offs.

### A life-history framework for the war on two fronts

Predators and parasites are a war on two fronts for prey/hosts. Intuitively, trade-offs ought to be prevalent, but life-history theory has illustrated that they will only be detectable under certain conditions. Specifically, the so-called Y-model of van Noorwijk and de Jong (1986) demonstrates how negative (trade-offs) or positive correlations between traits may arise depending on variation in resource acquisition and allocation (van Noorwijk and de Jong 1986; de Jong and Van Noordwijk 1992). Only when variation in allocation is larger than variation in acquisition does the model predict a trade-off between traits. Box 1 illustrates in more detail how the ratio of variation in acquisition to variation in allocation together determine the “space” within which relationships among trait values will vary.

**Box 1. The Y model depicts the rate at which genotypes acquire resources, and how they allocate these resources to different life history traits. In (a) the amount of resources acquired is represented by a, and the amount dedicated to Trait 1 is b**

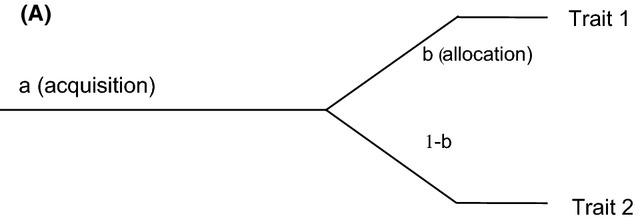

Variation in acquisition relative to allocation among genotypes predicts the sign of the genetic correlation, and thus the occurrence or not of trade-offs. In (b) and (c) we envision traits linked to parasites (*y*-axis) and predators (*x*-axis). Specifically, when there is very little variation in resource acquisition, substantial variation in allocation leads to a negative covariance between traits, as expected for traits involved in a trade-off (b). If, however, acquisition also varies, (because some individuals in a population are able to acquire more resources than others), but allocation varies relatively little, this effect can lead to a positive correlation between these traits when measured across individuals in a population, even while there is a resource-based trade-off within individuals (c).

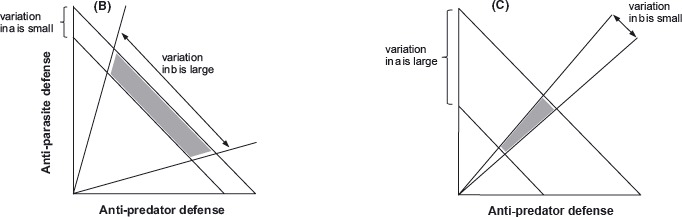

**Figure 1:**
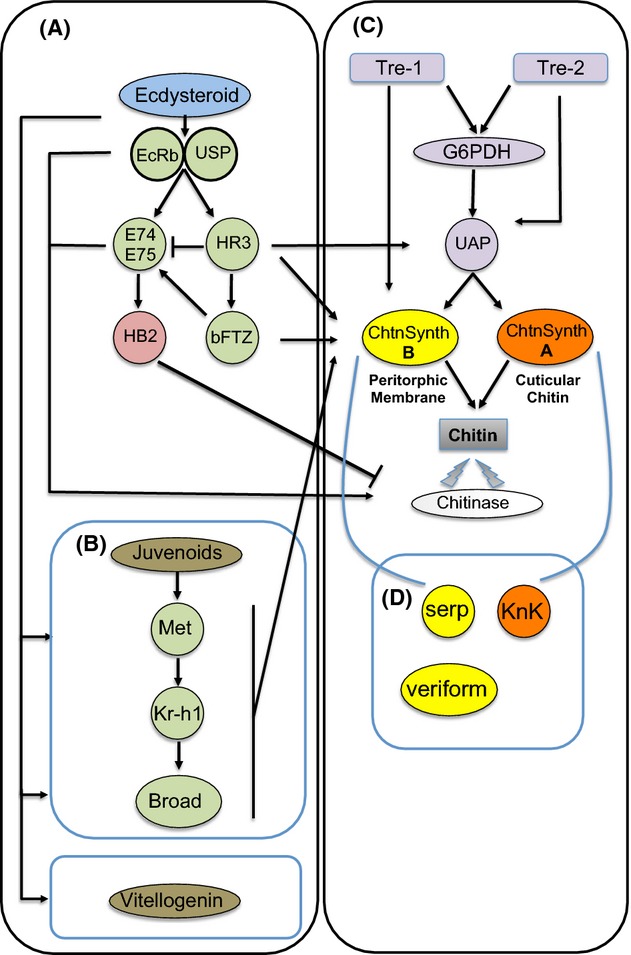
Candidate gene network regulating chitin synthesis and tying it to life-history variation via molting and immune function. (A, B) a set of highly conserved nuclear receptors (green) centered on ecdysone (EcRb-USP [RXR] network) and juvenile hormone signaling (Met - Kr-h1 – Broad) form a high-level control center for molting and maturation. We note that these two cascades are also tied to vetellogenin synthesis and the onset of reproduction. (C) This high-level control center is tied to the well-defined chitin synthesis pathway (purple), culminating in a synthesis bifurcation where cuticular chitin synthesis is likely tied to chitin synthase A and peritrophic membrane chitin is tied to chitin synthase B. (D) This is further supported by evidence that there are several genes/proteins linked specifically to either carapace or to tube-like anatomical features and peritrophic membrane structures (serp, veriform, and knk). Thus, these hormones, the nuclear receptors, and the chitin synthesis pathway ending in a split to carapace or gut chitin, form a hypothesized physiological backbone of any response to physical and temporal changes in chitin-rich structures. This putative and growing gene network should underpin the wide variety of predator and parasite-induced phenotypic plasticity we see throughout the arthropods. See text for details and references.

We propose an extension of the acquisition-allocation paradigm in upstream-downstream cellular and molecular events (d), here detailed with respect to the activation of the endocrine cascade that results in the expression of alternate chitin synthases (see Fig. 1). Here, the upstream processes are represented by a series of ligand-receptor binding events and in general the process of pathway activation. We suggest that this be analogous to resource acquisition. Following a fork in signal transduction, alternate stimulation of one of two downstream physiological processes is then analogous to allocation to one of two life history traits.

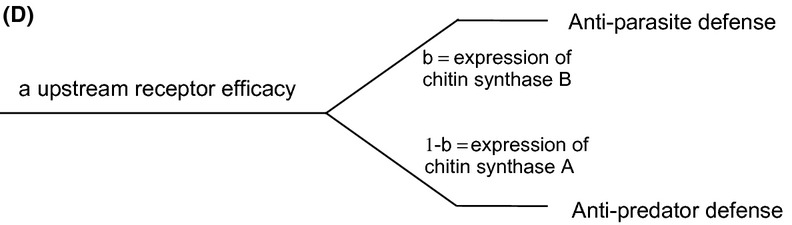

It follows that when there is relatively large variation in upstream receptor binding and activation efficacy, and relatively little variation in allocation to the two different possible outputs, a positive correlation between the strength of these two outputs may be evident. On the other hand, where there is relatively little variation in upstream receptor binding efficacy, and substantial variation in how individuals allocate to the two different physiological outputs, a trade-off may be evident. Coming at it the reverse way, a negative genetic correlation might well indicate that genetic variation occurs in the tines (and the genetic control of the two traits is not identical), whilst a positive genetic correlation would indicate that most variation occurs in the fork handle, and thus there is shared genetic control. Our extension captures specific hypothesis about gene expression that can thus challenge the qualitative predictions about shared genetic control from quantitative genetics.

One way to test these predictions and elucidate patterns of allocation and acquisition involves extending the hypothesis to consider the molecular/physiological control of allocation and acquisition (Zera and Zhao 2006). This requires three fundamental pieces of data: (1) a well-defined, potential trade-off, (2) a well-defined model of allocation, and (3) a well-defined set of genes that underpin the physiological control of allocation and the ensuing trade-off. We now offer such detail for shared defenses against predators and parasites, centered on the endocrine regulation of synthesis and degradation of chitin, a central structural component of arthropod body plans (Wigglesworth 1970; Jolles and Muzzarelli 1999). We then establish a novel link between this endocrine control hypothesis and life-history theory, revealing a physiological description of acquisition and allocation of resource in the context of shared defenses.

## Why Chitin? – The Physiological Basis of Defenses

From an ecological perspective, organisms might trade off investing into defense against predators and against pathogens. With our focus on arthropods, we propose that defenses to predators and parasites share a common physiological and genetic basis: the regulation of chitin synthesis and degradation. There are several well-established prey/host responses under predation risk/parasitism that involve some kind of physical or temporal modification of chitin-rich structures.

First, predation risk and parasitism alter life histories, specifically changing the time of, and size at maturity (Thornhill et al. 1986; Boersma et al. 1998; Riessen 1999; Tollrian and Harvell 1999; Krist 2001; Ebert et al. 2004; Chadwick and Little 2005; Beckerman et al. 2010). Such life-history changes, in arthropods, can only be realized by changing molt number and duration (Beckerman et al. 2010). Second, predators induce morphological alterations, including *de novo* or allometric changes which again implicate molting and chitin (Tollrian and Harvell 1999; Lass and Bittner 2002; Oda et al. 2011). The best known among these are the predator-induced helmets and neckteeth of the crustacean *Daphnia*. Similarly, the peritrophic membrane of arthropod guts is also chitin-rich, and this barrier is a major line of defense against pathogens and parasites (Dinglasan et al. 2009). Furthermore, central features of the arthropod innate immune system, such as the phenoloxidase (PO) cascade, melanization, and antimicrobial peptides (Soderhall and Cerenius 1998; Bulet et al. 1999) can be linked to chitin metabolism. Many antimicrobial peptides are chitin-binding and are tightly affiliated with chitinases (Tran et al. 2011), and the PO cascade may be itself triggered by chitin-binding antimicrobial peptides on chitinous scaffolds (Nagai et al. 2001). Finally, recent research has linked melanin, a key feature of invertebrate immune systems, to chitin metabolism (Marmaras et al. 1996; Walker et al. 2010).

### How does it work? Physical and temporal modification of chitin-rich structures

Both molting, gut development and features of the immune system are thus highly dependent on the ability to process and shape or remodel chitinous structures (Wigglesworth 1970; Jolles and Muzzarelli 1999; Merzendorfer and Zimoch 2003; Bauman and Wilson 2011). The developmental physiology of molting, transitions to maturity, and structural development across the Arthropoda can be framed in the signal cascade responsible for chitin synthesis; the entire process centers on ecdysone and juvenile hormone (JH) signaling (Fig. 1). Ecdysone is responsible for molting, whereas JH inhibits maturation to adulthood. A set of highly conserved nuclear receptor/transcription factor genes (Fig. 1A and B) form the high-level control center around ecdysone and JH signaling. It is increasingly acknowledged that regulatory networks comprising different combinations of these genes may be stage- or tissue-specific and that variation in these complexes, driven by the response to JH or ecdysone, or both, can determine cross-regulation of hormone activity (Bauman and Wilson 2011).

In the context of our hypothesis, the chitin synthesis and degradation pathway has two interesting properties: (1) it is a downstream target of the high-level control center for molt regulation focused around ecdysteroids and juvenile hormones (Fig. 1A–C) and (2) there are two classes of chitin synthases, A and B, produced at the end of the pathway. In *Tribolium castaneum,* cuticular chitin synthesis is tied to chitin synthase A, while peritrophic membrane chitin synthesis is tied to chitin synthase B (Arakane et al. 2005; Muthukrishnan et al. 2007; Khajuria et al. 2010). There are also several genes/proteins linked specifically to either cuticular structures or peritrophic membrane structures (Fig. 1D; Muthukrishnan et al. 2007; Chaudhari et al. 2011). These links emerge largely in the context of understanding the diversity of arthropod life cycles and center around interactions between ecdysone and JH management of the two forms of Chitin Synthase and the Broad-Complex, Met, and Kruppel-Homolog (Zhou et al. 1998; Zhou and Riddiford 2001, 2002; Erezyilmaz et al. 2005, 2006, 2009; Minakuchi et al. 2008).

Thus, the literature suggests that the (1) juvenoid and ecdysteroid hormones; (2) a highly conserved set of nuclear receptors, and (3) the chitin synthesis pathway ending in a split to carapace or gut chitin, together control responses to stress centered on temporal or spatial modifications of chitin-rich structures (Fig. 1). This putative and growing gene network should underpin the wide variety of predator- and parasite-induced phenotypic plasticity in chitin-rich structures we see throughout the arthropods. In the next two sections, we review evidence directly implicating aspects of this cascade (Fig. 1) in defenses to predators and parasites.

#### Chitin and predation

There is now evidence for the role of juvenile hormone and ecdysteroids in predator-induced defenses. Dennis et al. (unpubl. ms.) have shown that expression patterns in a small gene network, comprised of four nuclear receptors (EcR-RxR heterodimer, E75, HR3) and hemoglobin (HB2; see Fig. 1), can reveal whether the juvenile hormone or ecdysone pathway is regulating the morphological response to predation. Specifically, RT-qPCR experiments reveal that this network captures juvenile hormone regulation of morphological changes induced in early life stages of the crustacean *Daphnia pulex* by the invertebrate predator *Chaoborus* (Dennis et al. unpubl. ms.). Oda et al. (2011) have also shown that exposure of the arthropod *D. pulex* to methyl farnesoate – the crustacean juvenile hormone – can elicit an equivalent level of induced morphology that predator chemical cues do, directly implicating juvenoids in the induction of chitin-rich predator morphological defenses.

Juvenile hormone is central to the expression of many other morphological changes in arthropods. Topical application of juvenile hormone at critical times in beetle development gives rise to the fighting horned male morphs in *Onthophagus spp* and may be central to numerous exaggerated traits in insects (Emlen and Nijhout 1999, 2000). Ecdysone and juvenile hormones are also implicated in several other aspects of morphology and development including the control of color polymorphism in butterfly wings (Rountree and Nijhout 1995), temperature regulation of color morph in *Manduca sexta* (Truman et al. 1973), butterfly wing patterning (Brakefield et al. 1998), and temperature-dependent sex determination in *Daphnia* (Oda et al. 2005; Olmstead and LeBlanc 2007). Thus, ecdysteroid and juvenoid signaling, tied to chitin metabolism, play an important role in arthropod morphology generally and in predator-induced morphological and life-history defenses specifically.

#### Chitin and parasitism

Chitin and chitinases have been associated with host immune responses across a diverse group of organisms, ranging from plants (Tiffin and Moeller 2006) to arthropods (Shi and Paskewitz 2004; Kramer and Muthukrishnan 2009), and even mammals (in particular, chitinase-like proteins Nair et al. 2003; Lee et al. 2008). The peritrophic matrix, a chitinous layer in the midgut of arthropods, provides both structural and immune defense against parasites that invade through the gut (Lehane 1997). Juvenile hormone and ecdysone regulate chitin metabolism in the gut (Becker 1978) and regulate phenoloxidase (Flatt et al. 2008; Hiruma and Riddiford 2009). As the midgut is the point of entry for many arthropod parasites, this gut barrier, and immune activity there, forms an important first line of defense.

Gut-specific chitin synthases and chitinases have been found in several arthropod species (Kramer et al. 1993; Shen and Jacobs-Lorena 1997; Arakane et al. 2005; Dinglasan et al. 2009). The thickness of the peritrophic matrix is regulated by these gut chitinases (Filho et al. 2002), thereby creating a structural barrier to parasite entry. In addition, chitinases that cannot bind and degrade chitin have been identified in several arthropod species (Shi and Paskewitz 2004; Badariotti et al. 2007; McTaggart et al. 2009). Although their precise function remains to be determined, they may be immune-responsive and are probably most active in the midgut (Siva-Jothy et al. 2005).

These details, and the molecular network captured in Fig. 1, link a core endocrine regulated physiological process to defense in the context of multiple species interactions. They form the genetic and physiological basis for investigating whether and how a trade-off might arise when dealing with predators and parasites. Specifically, the data reveal a substantial list of “effectors” – genes that are upstream of the ultimate synthesis of chitin – and two products, Chitin Synthase A and B, which are linked, respectively, to gut and carapace chitin synthesis. We make the case below that these effectors and products form, respectively, a physiological description of acquisition (effectors) and allocation (products) of chitin resource in the context of shared defenses (Y-model; Box 1). This, we argue, forms the genetic basis of potential trade-offs between predator and parasite defenses.

### The hypothesis revisited – the war on two fronts

Our chitin-centered biological hypothesis presents a novel opportunity to explore many aspects of defense against alternate enemies and the types of trade-offs (or absence) that can be defined by a common, biochemical foundation. To translate the Y-model framework (acquisition and allocation), and its links to quantitative genetics (genetic correlations), into one more explicit in physiology and genes – the actual mechanisms underpinning life-history variation – requires connecting the abstract terms of acquisition and allocation to concrete features of physiology (Flatt and Heyland 2011). We propose a mechanization (*sensu* Stearns 1989) of the acquisition–allocation paradigm in upstream versus downstream cellular and molecular events (Box 1d).

In particular, we consider the network of upstream cell membrane receptors (ecdysone, juvenile hormone, Broad-complex) regulating one of two downstream physiological responses (gut or carapace chitin; Fig. 1A–D) as the embodiment of an endocrine cascade that ultimately terminates in the stimulation of one of two downstream physiological products. It is this “upstream” endocrine cascade, comprised of potentially several ligand-receptor activation events (signals), that is analogous to resource acquisition. Following a fork in signal transduction, one then expects a stimulation of one of two downstream physiological products; and this is analogous to allocation (of the signals) to one of two life-history traits (Box 1).

Thus, everything “above” chitin synthase A and B is upstream and essentially acquisition, and the “fork” at this point the allocation decision. The Y-model is thus generalizable to core physiology, and predictions about how variation in acquisition and allocation determine negative and positive genetic correlations are now extendable to molecular data. When there is relatively large variation in upstream (acquisition) receptor binding and activation efficacy, and relatively little variation in allocation to the two different possible outputs, a positive correlation between the two traits/end points will be expected. Conversely, where there is relatively little variation in upstream receptor binding efficacy, and substantial variation in how individuals allocate to the two different physiological outputs, a trade-off may be evident.

Thus, in the ecological context of predator and parasite defenses, when there is relatively large variation in upstream receptor binding and activation efficacy, and relatively little variation in allocation to predator versus parasite chitin synthase targets, a positive correlation is expected between traits defining the two defenses. On the other hand, where there is relatively little variation in upstream receptor binding efficacy, and substantial variation to the two chitin synthase targets, a trade-off is expected.

Thus, we are proposing a physiological generalization and extension of the quantitative genetic/Y-model framework for trade-offs. By tying life-history theory (Box 1) to a specific physiological hypothesis (Fig. 1) about the central role of chitin in the control of predator and parasite defenses, we offer an example that integrates modern molecular biology with community ecology. Our framework and hypothesis make it possible to do more than simply estimate genetic correlations and predict genetic control: endocrine-genetic detail provides target genes on which to assay variation in expression levels in the signaling and allocation cascade that defines the molecular control of the phenotype.

## Conclusion

Plant biologists have long embraced a philosophy of linking the expression of induced traits to both their environmental triggers and their developmental control (Baldwin et al. 2001; Kessler and Baldwin 2002; Wu and Baldwin 2010). We suggest here that there is space to apply this philosophy more widely. In fact, decades of developmental biology in arthropods points to the common and central regulation of defenses by one major endocrine physiological pathway: chitin synthesis and degradation, and thus, a single genetic pathway may allow an organism to defend against multiple threats. The life-history and acquisition/allocation framework, and in particular our genetic and physiological extension of the Y-model, suggests a valuable tool for exploring this hypothesis. We suggest that our framework should be generalizable to many situations where knowledge of a biochemical/endocrine pathway can be linked to particular phenotypes. The linking of the quantitative genetic study of life-history traits with their physiological and genetic underpinnings now holds, we believe, considerable potential for evolutionary and molecular ecology.
